# Coherence evaluation and first demonstration of multi-contrast X-ray computed tomography on NanoTerasu BL09W with an X-ray Talbot interferometer

**DOI:** 10.1107/S1600577526000512

**Published:** 2026-02-18

**Authors:** Ryosuke Ueda, Xiaoyu Liang, Chika Kamezawa, Hiroki Sumiishi, Yui Bishago, Takeyasu Nishio, Junya Yoshida, Masaharu Daimon, Shozo Hiramoto, Wolfgang Voegeli, Tetsuroh Shirasawa, Patrik Vagovič, Hiroyuki Yamane, Tetsuya Nakamura, Wataru Yashiro

**Affiliations:** a International Center for Synchrotron Radiation Innovation Smart (SRIS) Tohoku University Katahira 2-1-1, Aoba-ku Sendai980-8577 Japan; b Institute of Multidisciplinary Research for Advanced Materials (IMRAM) Tohoku University Katahira 2-1-1, Aoba-ku Sendai980-8577 Japan; c Graduate School of Engineering Tohoku University 6-6 Aramaki Aza Aoba, Aoba-ku Sendai980-8579 Japan; d Photon Science Innovation Center (PhoSIC), Aobayama Universe 306, 468-1 Aoba, Aramaki, Aoba-ku, Sendai980-0845, Japan; e Natural Sciences Division Tokyo Gakugei University 4-1-1 Nukuikita-machi, Koganei Tokyo184-8501 Japan; f National Institute of Advanced Industrial Science and Technology (AIST) Research Institute for Measurement and Analysis Instrumentation 1-1-1 Higashi Tsukuba Ibaraki305-8565 Japan; g European XFEL Holzkoppel 4 22869Schenefeld Germany; h Center for Free-Electron Laser, Notkestraße 85, 22607Hamburg, Germany; i Department of Applied Physics, Graduate School of Engineering The University of Tokyo 7-3-1 Hongo, Bunkyo-ku Tokyo113-8656 Japan; RIKEN SPring-8 Center, Japan

**Keywords:** X-ray Talbot interferometry, beam coherence, fourth-generation synchrotrons, X-ray phase-contrast imaging

## Abstract

We report on the coherence evaluation of the beamline BL09W at NanoTerasu (Tohoku University, Japan) and demonstrate X-ray multi-contrast imaging using an X-ray Talbot interferometer. Fringe visibility was measured in both the horizontal and vertical directions and analyzed to estimate the effective source size of the X-ray beam. The estimated source size in the horizontal direction was consistent with the design specification, confirming the validity of the measurements. In the vertical direction, the high visibility, exceeding 70%, indicates the remarkably high spatial coherence of the source. Multi-contrast computed tomography was successfully performed, simultaneously reconstructing absorption, phase and small-angle scattering contrasts. These results establish BL09W as a versatile beamline for coherence-based X-ray imaging.

## Abstract 2

Coherence properties were evaluated and multi-contrast X-ray computed tomography was demonstrated using an X-ray Talbot interferometer on the NanoTerasu beamline BL09W. The results confirm the high spatial coherence and imaging performance of the source, paving the way for advanced X-ray phase imaging.

## 1. Introduction

Fourth-generation synchrotron radiation facilities are designed to provide X-ray beams with remarkably high brilliance and spatial coherence, thereby enabling advanced applications in imaging and spectroscopy. NanoTerasu, a recently launched 3 GeV synchrotron light source based on a compact multibend achromat (cMBA) lattice (Obara *et al.*, 2025 ▸), is a representative example of such facilities. With a target emittance below 1 nm rad, it offers strong potential for coherence-based techniques, particularly X-ray phase-contrast imaging, which exploits the coherence of the beam.

X-ray phase-contrast imaging techniques have been extensively developed over the past few decades. Compared with conventional absorption-based imaging, they provide superior sensitivity, particularly for specimens composed of light elements. Several implementations have been proposed, including diffraction-enhanced imaging (Davis *et al.*, 1995 ▸), crystal interferometry (Momose, 1995 ▸; Yoneyama *et al.*, 2023 ▸), shearing interferometry (David *et al.*, 2002 ▸) and propagation-based methods (Wilkins *et al.*, 1996 ▸) that also enable single-image phase retrieval (Paganin *et al.*, 2002 ▸). These methods visualize the phase shifts induced as X-rays traverse a specimen, thereby revealing the spatial distribution of the refractive index. Recent developments have introduced alternative strategies for X-ray phase imaging, notably speckle-based (Berujon *et al.*, 2012 ▸; Zanette *et al.*, 2014 ▸; Zdora, 2018 ▸; Qiao *et al.*, 2020 ▸) and coded-aperture techniques (Olivo & Speller, 2007 ▸; Qiao *et al.*, 2021 ▸). These methods benefit from optically simple components, such as diffusers or masks, and are adaptable to various experimental conditions. However, they often involve trade-offs in phase sensitivity and noise robustness, and typically require complex data processing.

Among the various implementations, X-ray Talbot interferometry has been widely investigated as a representative technique for X-ray phase-contrast imaging (Momose *et al.*, 2003 ▸; Pfeiffer *et al.*, 2006 ▸; Weitkamp *et al.*, 2005 ▸; Weitkamp *et al.*, 2006 ▸; Momose, 2005 ▸). It performs effectively even with polychromatic sources and allows relatively straightforward implementation over large fields of view, making it attractive for applications ranging from medical imaging to materials science. The visibility of interference fringes in a Talbot interferometer depends strongly on the spatial coherence of the incident beam, and higher coherence leads to improved visibility. Because the signal-to-noise ratio of the reconstructed images increases with visibility, the remarkably high brilliance and spatial coherence of fourth-generation synchrotron sources are expected to enable X-ray phase-contrast imaging with high sensitivity and improved image quality.

Beyond imaging applications, Talbot interferometry has also been applied to characterize the spatial coherence of X-ray beams. Early studies demonstrated that fringe visibility in fractional Talbot imaging reflects the transverse coherence length (Cloetens *et al.*, 1997 ▸; Guigay *et al.*, 2004 ▸) and subsequent work developed the method into a quantitative tool for source characterization under monochromatic illumination (Marathe *et al.*, 2014 ▸; Shi *et al.*, 2025 ▸).

In this study, we investigate the spatial coherence of the BL09W beamline at NanoTerasu using an X-ray Talbot interferometer. Visibility measurements were performed to estimate the effective source size, providing a benchmark for the current beamline optics. While previous coherence evaluations have mainly employed monochromatic configurations, our implementation is based on a polychromatic beam without monochromators. We also demonstrate multi-contrast X-ray computed tomography, simultaneously reconstructing absorption, phase and scattering contrasts. These results highlight the practical imaging capabilities enabled by the coherence of NanoTerasu and provide a foundation for future developments in high-sensitivity X-ray imaging.

## 2. BL09W beamline at NanoTerasu

**Figure 1:**
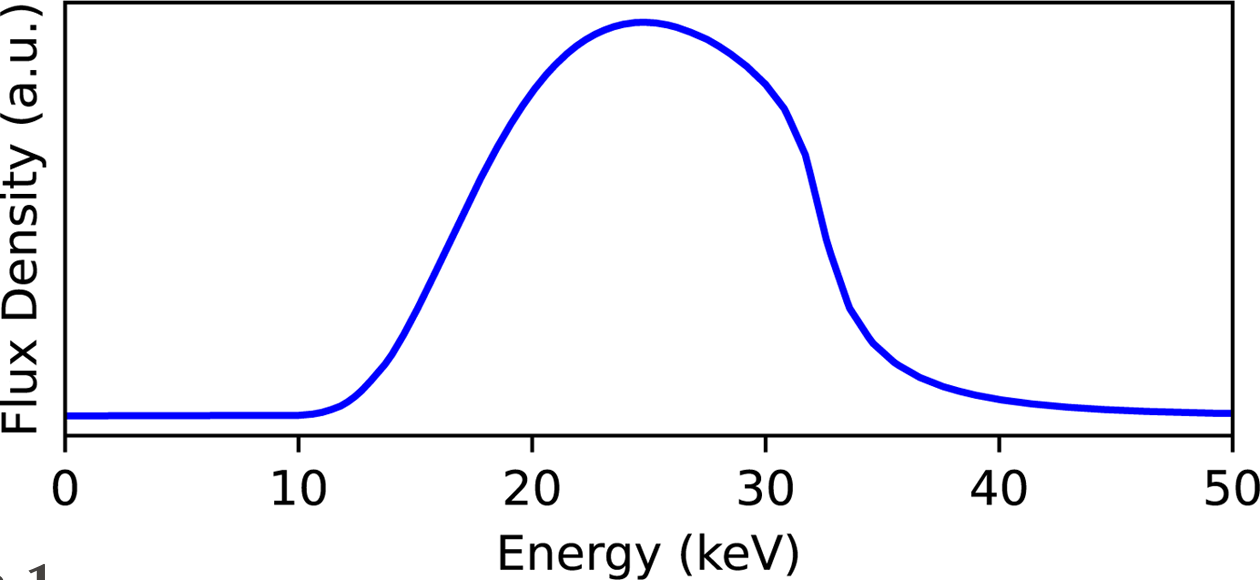
Energy dependence of the X-ray flux density on beamline BL09W, calculated using the simulation software *SPECTRA*. The result includes the effects of platinum mirror reflectivity and a 0.3 mm thick beryllium filter.

NanoTerasu is a fourth-generation 3 GeV high-brightness synchrotron radiation facility located on the Aobayama new campus of Tohoku University in Sendai, Japan. It began operation in April 2024. BL09W is a white X-ray beamline based on a multipole wiggler (120 mm × 5 periods) that delivers a continuous spectrum from 4 keV to 30 keV (photons below 4 keV are suppressed by a beryllium window and those above 30 keV are cut by a flat platinum mirror; the spectrum exhibits a maximum near 25 keV). The expected flux density calculated using the simulation software *SPECTRA* (Tanaka & Kitamura, 2001 ▸) is shown in Fig. 1 ▸. The designed source size is 81 µm (horizontal, H) × 2 µm (vertical, V). A white X-ray beam is reflected at an incident angle of 2.6 mrad by a platinum-coated plane mirror and introduced into the experimental hutch, which is located about 25 m downstream. The maximum beam size at the sample position is approximately 50 mm (H) × 4 mm (V) with a source-to-sample distance of approximately 54 m. Owing to the large beam size and high flux, BL09W is well suited to high-speed computed tomography (CT) and X-ray phase-contrast imaging.

**Figure 2:**
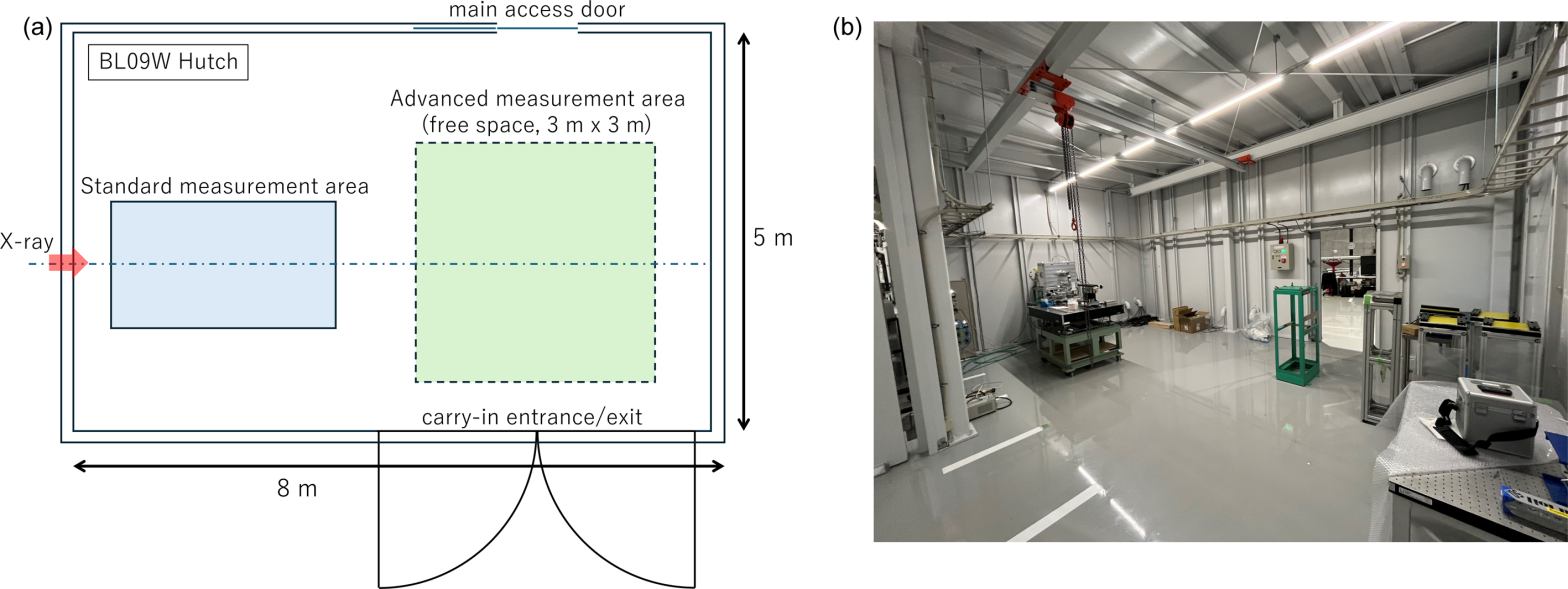
Layout of the experimental hutch on the NanoTerasu beamline BL09W. (*a*) Schematic layout of the experimental hutch. (*b*) Photograph taken from the carry-in door.

The layout of the BL09W experimental hutch and a photograph taken from the carry-in door are shown in Figs. 2 ▸(*a*) and 2 ▸(*b*), respectively. The hutch measures 8 m along the optical axis, 5 m in width and 4 m in height. A standard white X-ray CT setup is installed in the upstream part of the experimental hutch, while the downstream section provides a 3 m × 3 m free space for user-supplied apparatus. This free space allows advanced measurements not supported in the standard setup, such as 4D-CT (Yashiro *et al.*, 2017 ▸; Yashiro *et al.*, 2018 ▸; Mashita *et al.*, 2021 ▸; Mashita *et al.*, 2023 ▸). The downstream free space also enables flexible placement of detectors, optical components and sample manipulators, providing versatility for developing advanced techniques, including multi-beam CT (Yashiro *et al.*, 2022 ▸; Voegeli *et al.*, 2022 ▸; Liang *et al.*, 2023 ▸; Voegeli *et al.*, 2023 ▸; Sumiishi *et al.*, 2024 ▸; Yashiro *et al.*, 2024 ▸; Shirasawa *et al.*, 2025 ▸).

In this study, a Talbot interferometer was installed in the free space of the hutch. The beam properties, particularly the source size, were evaluated via visibility measurements. Multi-contrast CT was also performed to demonstrate the X-ray phase imaging capability.

## 3. Visibility in Talbot interferometry

### 3.1. Fringe visibility and spatial coherence

**Figure 3:**
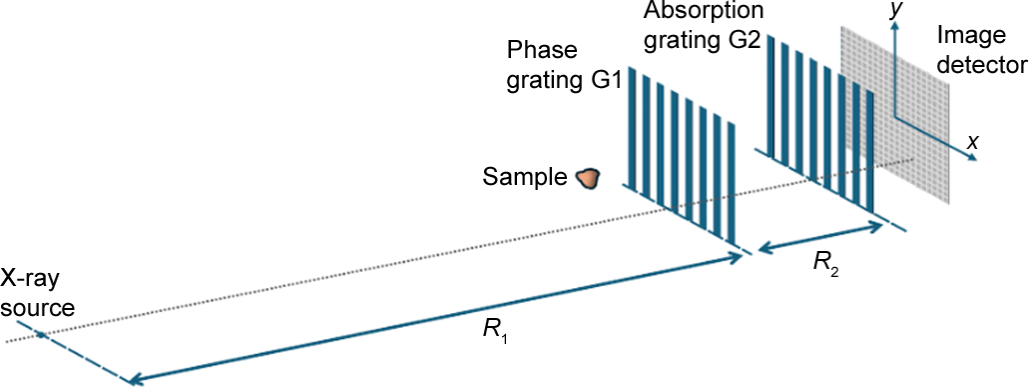
Schematic layout of the Talbot interferometer.

A typical setup of a Talbot interferometer is shown in Fig. 3 ▸. In a Talbot interferometer, the G1 phase grating generates a spatially modulated intensity pattern downstream as a result of interference between X-rays transmitted directly through the grating and those diffracted by the grating.

Interference occurs when the grating period is shorter than the spatial coherence length of the incident X-rays. Therefore, diffraction gratings with periods of several micrometres are typically used. The G1 grating produces a periodic self-image downstream, replicating the grating pattern. Since the self-image period is comparable to that of the grating, a high-resolution detector is required to resolve it, but such detectors generally have a limited field of view. To overcome this limitation, a G2 absorption grating is placed at the self-image plane, and the long-period moiré pattern generated by the interference between the self-image and the G2 grating is recorded. The contrast of the fringes, referred to as the visibility, increases with the spatial coherence length of the X-rays.

The spatial coherence length *L*_c_ is given by 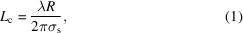
where λ is the X-ray wavelength, *R* is the distance from the source and σ_s_ is the source size (Yashiro *et al.*, 2008 ▸). From equation (1), the spatial coherence length is inversely proportional to the source size. Therefore, the source size can be estimated from the visibility measured in a Talbot interferometer. In the following, we describe the effect of source size on visibility from the perspective of Fresnel integrals.

### 3.2. Wave propagation model for visibility calculation

Let *R*_1_ denote the distance from the source to the G1 phase grating and *R*_2_ the distance from G1 to G2. The detector plane is located immediately downstream of G2. Numerous studies have reported methods for wave propagation calculations (Paganin *et al.*, 2002 ▸; Yashiro *et al.*, 2010 ▸; Shanblatt *et al.*, 2019 ▸; Ueda & Momose, 2023 ▸). In this study, we formulate the propagation using the Wigner distribution (Bastiaans, 1986 ▸; Yan *et al.*, 2018 ▸), which is particularly suitable for incorporating coherence effects.

The Wigner distribution *W*(*x*, *u*) describes the behavior of waves in the phase space (*x*, *u*), where *x* is the position and*u* is the spatial frequency perpendicular to the propagation direction. By integrating over frequency, the intensity *I*(*x*) is obtained as 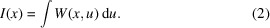
The Wigner distribution can be expressed as the Fourier transform of the mutual intensity *J*,
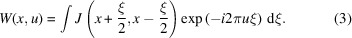
The mutual intensity *J*(*x*_1_, *x*_2_) is a physical quantity that describes the correlation (coherence) of the wavefield at positions *x*_1_ and *x*_2_, and it is closely related to the spatial distribution of the source. When the source has a finite spatial extent, *J*(*x*_1_, *x*_2_) decreases with increasing separation between *x*_1_ and *x*_2_, reflecting the effect of the source size. For example, for an incoherent source with a Gaussian profile of standard deviation σ_s_, the mutual intensity *J*(*x*) is given by the Van Cittert–Zernike theorem (Goodman, 1985 ▸; Born & Wolf, 1993 ▸) as 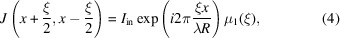

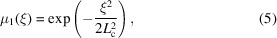
where μ_1_(ξ) represents the coherence function describing the decay in the correlation with increasing separation ξ. As the source size increases, the coherence between different positions decreases, leading to reduced visibility observed in the Talbot interferometer.

The Wigner distribution immediately downstream of a grating is obtained from the incident mutual intensity and the transmission function of the grating. For the G1 grating, it can be expressed as 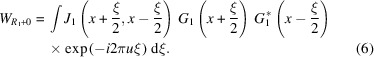
Since diffraction gratings are periodic, their transmission functions can be represented as Fourier series expansions, 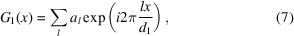

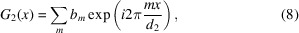
where *d*_1_ and *d*_2_ are the periods of gratings G1 and G2, respectively. The Wigner distribution after propagation over a distance *R*_2_ can be written as 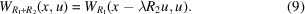
This property allows wave propagation to be described in a compact and convenient form.

By organizing equations (2)–(9), the intensity at the position (*x*, *R*_2_) just downstream of G2 can be expressed as 
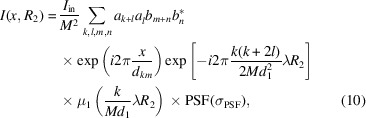
where *M* = (*R*_1_ + *R*_2_)/*R*_1_ and *d*_*km*_ = (*k*/*Md*_1_ + *m*/*d*_2_)^−1^. *M* is the magnification factor of the G1 grating at the G2 position and *d*_*km*_ represents the moiré period. When *Md*_1_ ≃ *d*_2_, a long-period moiré pattern generated by the G2 grating appears for |*k* + *m*| = 1, which can be observed by the imaging detector.

The blurring effect of the optical system is modeled by a point spread function (PSF), represented by a Gaussian function 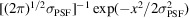. The corresponding factor is 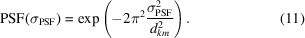
The intensity for polychromatic X-rays is obtained as a weighted average over the spectral distribution *S*(*E*),
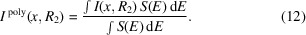
The spectral distribution *S*(*E*) was estimated using *SPECTRA* (Tanaka & Kitamura, 2001 ▸), taking into account the effects of the beryllium window, platinum mirror and detector efficiency. The detector efficiency was approximated based on the absorption characteristics of the scintillator. From the polychromatic intensity *I*^poly^(*x*, *R*_2_), the fringe visibility can be calculated as 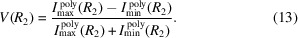


## 4. Experimental results

### 4.1. Visibility measurements

A Talbot interferometer as shown in Fig. 3 ▸ was installed in the downstream free space of the BL09W experimental hutch to quantify the fringe visibility as a function of grating separation.

The system consisted of a gold phase grating (G1) with a thickness of 2.7 µm, designed to provide a π/2 phase shift for 25 keV X-rays, and a gold absorption grating (G2) with a thickness of 60 µm. Both gratings had an identical period *d*_1_ = *d*_2_ = 5.3 µm. The source-to-G1 distance was *R*_1_ ≃ 54 m.

Moiré patterns were recorded while varying the G1–G2 distance *R*_2_ to evaluate the coherence. As indicated by equation (1), the visibility and spatial coherence depend on the source size. Because the source size is larger in the horizontal than in the vertical direction, the grating orientation was alternated to measure the visibility in both the horizontal and vertical directions.

Fringes were imaged using a 10 µm thick GAGG (Ce:Gd_3_Al_2_Ga_3_O_12_) scintillator coupled to a beam monitor (Hamamatsu Photonics AA40, lens focal length *f* = 50 mm) and an additional *f* = 200 mm lens, yielding a 4× magnification. A high-speed CMOS camera (Photron Fastcam NOVA S12, 1024 × 1024 pixels, 20 µm pixel size) provided an effective pixel size of 5 µm. The exposure time per frame was 1/12800 s.

**Figure 4:**
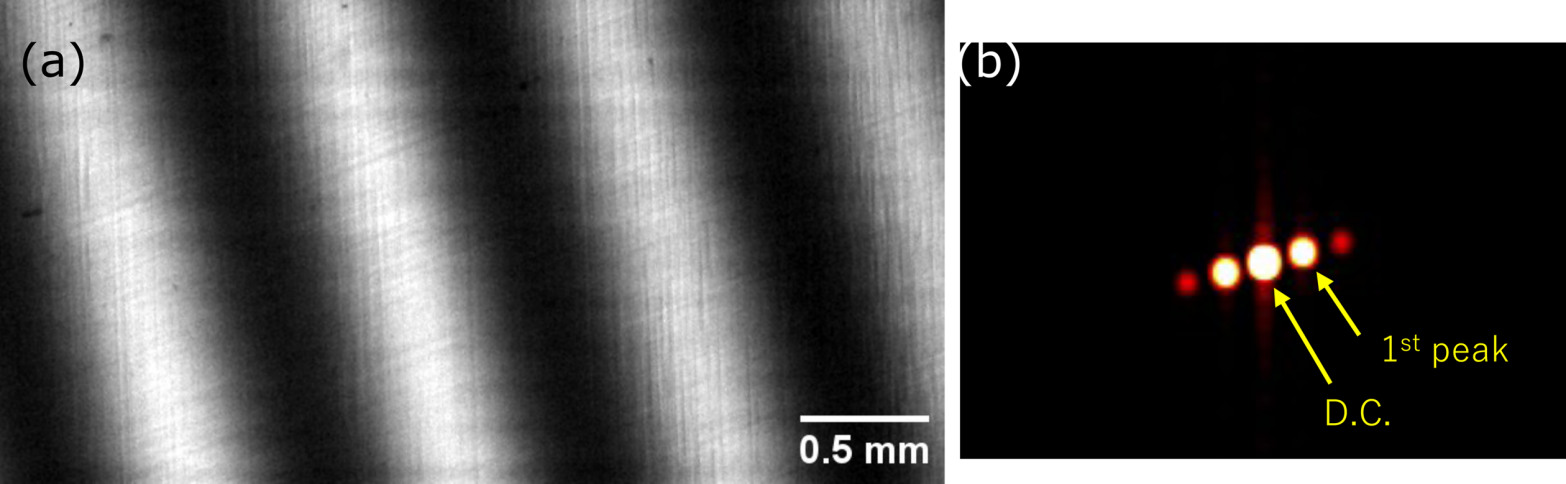
(*a*) Representative moiré fringes (*R*_2_ = 0.237 m, grating period horizontal). (*b*) Power spectrum (FFT). Visibility is derived from the DC and first-order peak amplitudes.

The visibility was extracted from each frame set at a fixed *R*_2_ using a Fourier analysis, where the modulus of the first-order moiré peak was normalized by the DC component. When the moiré period became long and spectral overlap between the DC and first harmonic degraded separability, zero-padding and a Blackman window were applied to stabilize peak localization. An example (with the grating period oriented horizontally, *R*_2_ = 0.237 m) is shown in Fig. 4 ▸; the clear separation of DC and first-order peaks corresponds to a visibility of ∼0.68. Note that the apparent tilt of the fringes in Fig. 4 ▸(*a*) is a rotational moiré and does not affect the visibility evaluation.

**Figure 5:**
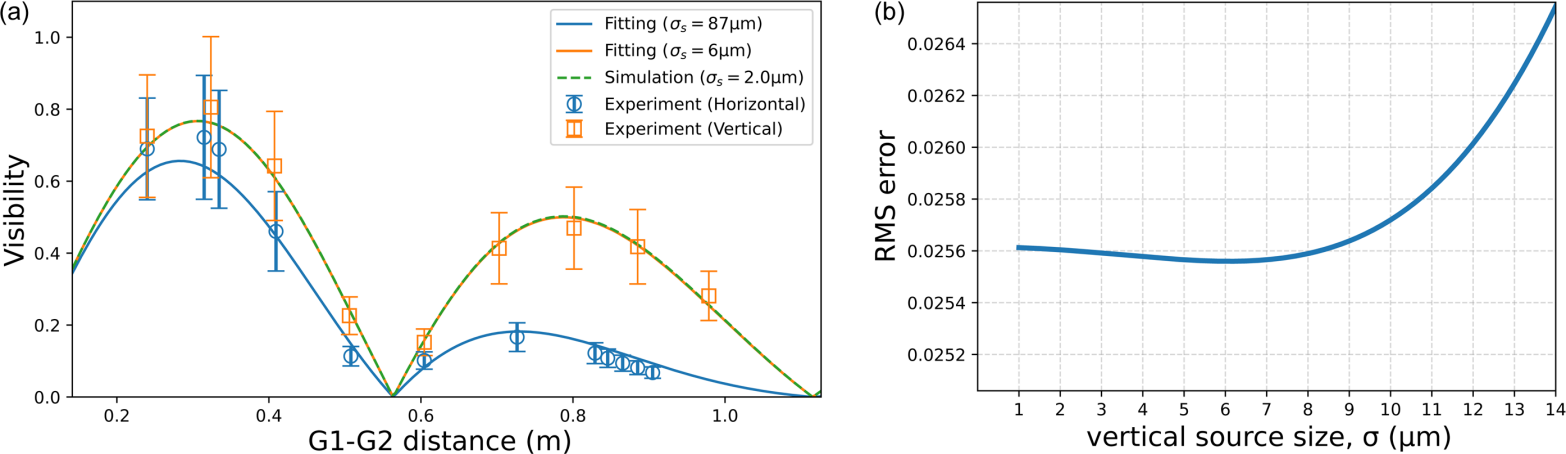
(*a*) Measured visibilities (circles and squares) as a function of the G1–G2 distance *R*_2_, together with the polychromatic model fits (solid curves). Estimated source sizes are σ_s,H_ = 87 µm and σ_s,V_ = 6 µm. The dashed curve represents the vertical visibility for σ_s,V_ = 2 µm. (*b*) RMS error between the measured and modeled visibilities as a function of vertical source size σ_s,V_.

Fig. 5 ▸(*a*) plots the measured visibility as a function of *R*_2_, with error bars representing the uncertainty in the Fourier peak position. In a Talbot interferometer, the self-image (and thus a visibility maximum) occurs at *R* = *pd*_1_*d*_2_/λ^2^, where *p* is the Talbot order. For a π/2 phase grating, the relevant sequence is *p* = 1/2, 3/2, 5/2,…, while the moiré contrast vanishes at *p* = 1. Our geometry targets the *p* = 1/2 maximum at *R*_2_ = 0.283 m. A secondary maximum at *p* = 3/2 is weakened because it requires a longer spatial coherence length. These characteristic features are well reproduced experimentally: near *p* = 1/2, the vertical visibility reaches ∼0.8 and the horizontal exceeds 0.6, consistent with the smaller intrinsic vertical source size and demonstrating high partial coherence even horizontally. For comparison, in a previous Talbot interferometry experiment conducted on the white X-ray beamline BL28B2 at SPring-8 using the same gratings, the measured visibility at *p* = 1/2 was approximately 0.4. This indicates that the spatial coherence of the NanoTerasu beamline BL09W is remarkably high compared with that of a typical third-generation synchrotron source.

To quantify the source size, the measured visibilities were fitted using the model given in equations (1), (5), (10) and (13), incorporating the polychromatic spectrum from *SPECTRA* and the scintillator efficiency. The fitted curves for the horizontal and vertical visibilities are shown in Fig. 5 ▸(*a*) (solid lines). For 25 keV X-rays, the nominal *p* = 1/2, 1 and 3/2 distances are *R*_2_ = 0.283 m, 0.566 m and 0.849 m, respectively; slight shifts in the observed extrema are attributed to spectral bandwidth. The estimated source sizes are σ_s,H_ = 87 µm (horizontal) and σ_s,V_ = 6 µm (vertical). The horizontal size is consistent with the design specification of 81 µm, whereas the vertical size is somewhat larger than the design value of 2 µm. The discrepancy in the vertical direction arises from the reduced parameter sensitivity in the high-visibility regime; in this regime, the visibility is more sensitive to the spectral distribution than to the source size. The visibility curve for σ_s,V_ = 2 µm is also shown in Fig. 5 ▸(*a*) (dashed line), and the two curves for σ_s,V_ = 2 µm and σ_s,V_ = 6 µm are nearly indistinguishable. Fig. 5 ▸(*b*) shows the root-mean-square (RMS) error between the measured and modeled visibilities as a function of the vertical source size. The RMS error does not vary significantly for σ_s,V_ < 10 µm. These results indicate that, although the source size in the vertical direction cannot be accurately quantified, it can be confidently estimated to be below 10 µm.

### 4.2. Multi-contrast CT with the Talbot interferometer

Talbot interferometry typically achieves higher fringe visibility under monochromatic illumination, while polychromatic conditions tend to reduce the visibility due to the broad spectral bandwidth. Nevertheless, the present white-beam configuration on BL09W achieved high visibility, reaching approximately 80% in the vertical direction and over 60% in the horizontal direction without the use of a monochromator. These results indicate the potential for achieving a higher signal-to-noise ratio than typically attainable under white-beam conditions at third-generation synchrotron facilities. The ability to perform such measurements under polychromatic illumination suggests that short-exposure acquisitions are practicably feasible, opening the way to improved image quality in high-speed applications, including 4D-CT. In this section, we demonstrate an application of Talbot interferometry to multi-contrast X-ray imaging. The interferometer setup, including gratings and detector, was identical to that described in Section 4.1. Here, we focus on the additional experimental conditions required for CT acquisition and on the resulting reconstructions, which demonstrate the simultaneous retrieval of absorption, phase and scattering contrasts (Pfeiffer *et al.*, 2008 ▸).

For data acquisition, continuous phase stepping was adopted (Kibayashi *et al.*, 2012 ▸; Yashiro *et al.*, 2018 ▸), in which one of the gratings was continuously translated while images were recorded. This approach is particularly effective for high-intensity white synchrotron radiation, as it enables rapid collection of multiple phase steps without mechanical interruptions. A total of 2000 projections were recorded per rotation, during which the grating was shifted by one phase step. With nine phase steps, corresponding to nine rotations, a complete CT dataset was acquired in only 1.8 s at 10000 frames s^−1^, with an exposure time of 0.1 ms per frame. Although image data were collected over a full 360° rotation, only 180° (1000 projections) were used for reconstruction.

The recorded frames were grouped into nine phase-step images and processed using a phase retrieval algorithm to obtain absorption, differential phase and small-angle scattering (dark-field) images. Various algorithms have been proposed for phase retrieval from fringe images (Vargas *et al.*, 2011 ▸; Kando *et al.*, 2019 ▸; Lian *et al.*, 2019 ▸; Lian *et al.*, 2021 ▸; Ueda & Momose, 2024 ▸). We employed a least-squares-based approach similar to that given by Wang & Han (2004 ▸), considering both the simplicity of the algorithm and slight deviations among the phase steps. The scattering image obtained with the grating interferometer is sensitive to the orientation of the grating period. By selecting the grating direction, anisotropy in the sample structure can be captured. In this experiment, the grating period was oriented horizontally, making the scattering image sensitive to microstructures oriented vertically, that is, perpendicular to the grating lines. Tomographic images were reconstructed using the filtered backprojection (FBP) method. The Shepp–Logan filter (Shepp & Logan, 1974 ▸) and the Hilbert filter (Pfeiffer *et al.*, 2007 ▸) were applied to the absorption and scattering images, and to the phase images, respectively.

**Figure 6:**
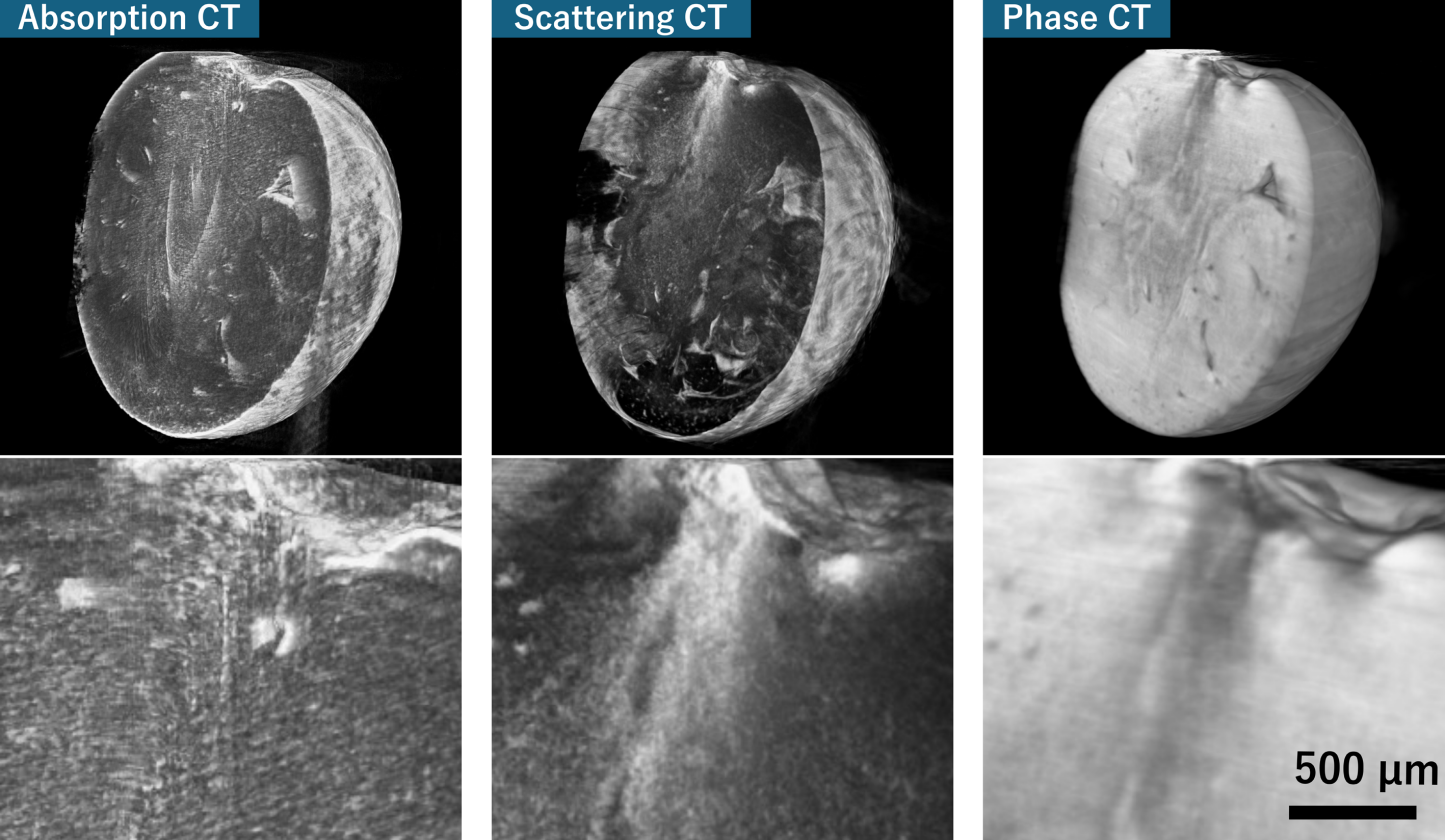
Volume-rendered multi-contrast X-ray CT reconstructions of a cherry tomato obtained with a Talbot interferometer. (Top row, left to right) Absorption, small-angle scattering (dark-field) and phase-contrast volumes. (Bottom row, left to right) The magnified vascular bundle region for each contrast, where the scattering and phase images clearly highlight fine fibrous vascular structures that are indistinct in the absorption image.

A cherry tomato was used as the test specimen, and representative reconstructions are shown in Fig. 6 ▸. The absorption image clearly delineates regions of strong density contrast, such as the pericarp and seeds, but fine structures inside the pulp remain indistinct. In contrast, the phase image enhances weak refraction-induced variations, providing high-contrast visualization of internal boundaries and fibrous structures. The scattering (dark-field) image reveals additional signals arising from microscopic cellular features, complementing the absorption and phase modalities. Notably, the vascular bundles are clearly highlighted in the phase and scattering images, whereas they are difficult to identify in the absorption image.

These results demonstrate that multi-modal CT with a Talbot interferometer can exploit the high spatial coherence of a fourth-generation synchrotron source to provide high-sensitivity information-rich structural imaging within a remarkably short acquisition time (1.8 s per CT). The method is effective for nondestructive studies of soft tissues and low-*Z* materials (Momose *et al.*, 1996 ▸) and holds strong potential for future developments towards quantitative 3D analysis and time-resolved investigations.

## 5. Discussion

The visibility-based estimation of source size provided important insights into the spatial coherence properties of beamline BL09W. In the horizontal direction, the estimated source size was consistent with the design value, demonstrating that our approach is reliable for characterizing source properties. In contrast, in the vertical direction, the estimation exhibited a reduced sensitivity to the source size in the high-visibility regime, resulting in a slightly larger source size than the design value. We attribute this insensitivity to the polychromatic nature of the X-ray beam. To overcome this limitation, future experiments using an Si monochromator could suppress the wavelength dependence and enable a more precise evaluation of the intrinsic source size.

In addition, we note that the vertical coherence may also be affected by the platinum mirror placed upstream of the experimental hutch. The mirror is used for higher-order harmonic rejection, reflecting the beam at an angle of approximately 5 mrad in the vertical direction. Thus, the vertical coherence may be degraded by the mirror surface quality, such as roughness or figure errors (*e.g.* bending and slope variations). Similar effects have been discussed in previous studies (Yabashi *et al.*, 2001 ▸; Vartanyants & Robinson, 2003 ▸), which pointed out that vertical reflection tends to degrade spatial coherence in the vertical direction and that even well polished mirror surfaces can introduce partial decoherence. From the images obtained on beamline BL09W, the mirror surface quality is not excessively rough. Therefore, we consider that the order of magnitude of the estimated vertical source size is reasonable, although some degradation cannot be completely ruled out. In principle, the reduction in coherence by the mirror could be minimized by changing the reflection direction to horizontal. However, such a modification would require a major reconfiguration of the beamline and is therefore impractical under the current operating conditions.

Beyond the coherence evaluation, the present results demonstrate multi-contrast CT imaging using X-ray Talbot interferometry on beamline BL09W. Future developments are expected to include time-resolved phase imaging and 4D-CT, enabled by the high flux and spatial coherence, following recently conducted experiments at other synchrotron facilities (Mashita *et al.*, 2023 ▸; Ueda *et al.*, 2023 ▸). By combining single-shot phase retrieval algorithms (Kando *et al.*, 2019 ▸; Lian *et al.*, 2019 ▸; Ueda & Momose, 2024 ▸), which do not require phase stepping, we could further improve the temporal resolution of multi-contrast 4D-CT. The high degree of coherence also opens up opportunities for further advanced imaging. For instance, employing higher Talbot orders can enhance phase sensitivity, as demonstrated by Vila-Comamala *et al.* (2021 ▸). Such advanced imaging techniques will broaden the range of applications in biomedical and materials sciences, helping establish BL09W as a versatile beamline for coherence-based X-ray imaging.

## 6. Conclusion

With the advent of fourth-generation synchrotron radiation sources, the remarkably high brilliance and spatial coherence of their X-ray beams have opened new opportunities for advanced imaging techniques. The aim of this study was to investigate the spatial coherence of the NanoTerasu BL09W beamline using an X-ray Talbot interferometer and to demonstrate multi-contrast X-ray CT imaging.

From the visibility measurements, the estimated source size in the vertical direction was consistent in order of magnitude with the design, although a slight deviation was observed, probably due to the influence of the polychromatic spectrum. In the horizontal direction, the estimated source size was in good agreement with the design. These findings support the reliability of the interferometric approach for characterizing the source properties.

The successful demonstration of multi-contrast X-ray CT imaging highlights the capability of Talbot interferometry to exploit fully the high coherence of fourth-generation synchrotron radiation. These results provide a valuable basis for further optimization of beamline BL09W and point towards future developments in high-sensitivity and time-resolved X-ray phase imaging.

## Data Availability

The data that support the findings of this study are available within the article.
